# Cognitive Appraisals Affect Both Embodiment of Thermal Sensation and Its Mapping to Thermal Evaluation

**DOI:** 10.3389/fpsyg.2016.00800

**Published:** 2016-06-27

**Authors:** Trevor P. Keeling, Etienne B. Roesch, Derek Clements-Croome

**Affiliations:** ^1^BuroHappold EngineeringLondon, UK; ^2^School of Built Environment, University of ReadingReading, UK; ^3^Centre for Integrative Neuroscience and Neurodynamics, School of Psychology and Clinical Language Sciences, University of ReadingReading, UK

**Keywords:** emotion, appraisal dimensions, psychological adaption, thermal comfort, embodiment

## Abstract

The physical environment leads to a thermal sensation that is perceived and appraised by occupants. The present study focuses on the relationship between sensation and evaluation. We asked 166 people to recall a thermal event from their recent past. They were then asked how they evaluated this experience in terms of 10 different emotions (frustrated, resigned, dislike, indifferent, angry, anxious, liking, joyful, regretful, proud). We tested whether four psychological factors (appraisal dimensions) could be used to predict the ensuing emotions, as well as comfort, acceptability, and sensation. The four dimensions were: the Conduciveness of the event, who/what caused the event (Causality), who had control (Agency), and whether the event was expected (Expectations). These dimensions, except for Expectations, were good predictors of the reported emotions. Expectations, however, predicted the reported thermal sensation, its acceptability, and ensuing comfort. The more expected an event was, the more uncomfortable a person felt, and the less likely they reported a neutral thermal sensation. Together, these results support an embodied view of how subjective appraisals affect thermal experience. Overall, we show that appraisal dimensions mediate occupants' evaluation of their thermal sensation, which suggests an additional method for understanding psychological adaption.

## Introduction

### Thermal environment, thermal sensation, and evaluative response

Treating thermal comfort as a problem of *energy balance* lends itself to building design practices based on physiology. However, adaptive comfort theory (Nicol and Humphreys, 1973; de Dear and Brager, 1998) contributes scope for a range of *psychological factors* to be considered as well. It indeed seems intuitive that some part of thermal comfort involves the occupants' thermal expectations and preferences, and in turn may be constitutive of the overall experience (Clements-Croome, 2013). The aim of the present study is to reveal a mechanism whereby the thermal environment is perceived and internalized by occupants and to show that this evaluative process shapes thermal experience.

For the purpose of the present investigation, thermal experience is broken down into three components. First, physical environments, such as air temperature, air movement, etc., constitute the medium within which occupants operate. Secondly, thermal sensation is the interface between the occupant and the environment, which is predominately described using the ASHRAE thermal sensation scale, which runs from cold, through cool, neutral, warm, to hot (ASHRAE, 2010). Thirdly, an occupant's evaluation of their thermal environment can be used to describe the process of reflection upon the sensation. Conventionally evaluation criteria of satisfaction, comfort and acceptability are used.

We look at the psychological factors that shape how thermal sensations are perceived and evaluated, by grounding our investigation in the field of emotion psychology. Particularly, we are interested in the way that four psychological factors (“appraisal dimensions”) may shape the criteria of acceptability, comfort, thermal sensation, and the ensuing emotional experience, which result from the exposure to a particular thermal environment, or thermal event. We aim to render explicit the relationship between the occupant's psychology and their thermal experience, and hope to inform building design practices by situating occupants at the center of the space they occupy.

### Models of thermal comfort

**Figure 1:**
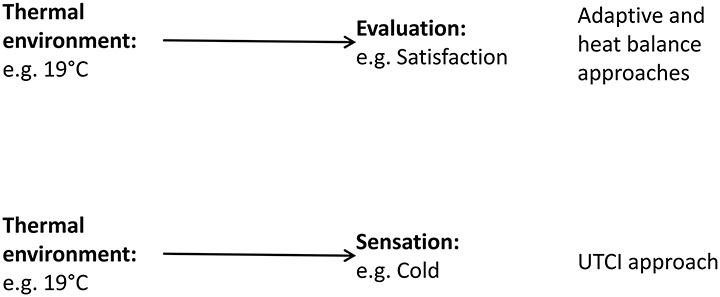
**Thermal models tend to focus on the thermal environment and either sensation or evaluation**. They tend to overlook the relationship between sensation and evaluation.

Models of thermal comfort attempt to predict evaluation or sensation dependent upon the physical environment. For instance, both the *energy balance* and *adaptive comfort* approaches relate the indoor thermal environment to evaluation of (satisfaction and comfort) (Fanger, 1970; de Dear and Brager, 2001; ASHRAE, 2010). The universal thermal climate index (UTCI) relates outdoor thermal environment to thermal sensation (Fiala et al., 2012). These theories focus on the relationship between thermal environment and either thermal sensation or thermal evaluation. In their basic usage, they overlook processes that map a person's thermal sensation to their thermal evaluation (Figure 1).

Physiological models of thermal experience describe the energy flows within the body. They split the body into several layered sections, each with different thermal properties, which are used to predict the energy balance and temperature throughout the body (Fiala et al., 2012; Schellen et al., 2013). Then by understanding these body temperatures and their rates of change, thermal sensation can be predicted (Fiala et al., 2012; Kingma et al., 2012). This still leaves the problem of relating a given thermo-physiological state to an evaluation of the thermal environment. Most often, the above-mentioned theories will assume that thermal neutrality is desired and equates to maximum comfort (Fanger, 1970).

Alliesthesia provides one explanation why a neutral thermal sensation, or any other single thermal sensation, will not always lead to the same evaluation. As such, it provides a theoretical approach to understanding the relationship between sensation and evaluation. It suggests that when a person is overheated they will find a cold sensation pleasant, whilst when a person is overcooled they will find a hot sensation pleasant (Cabanac, 2006; Parkinson and de Dear, 2015). However, alliesthesia relies on a physiological approach to explain the mapping between sensation and evaluation. In contrast, we aim to demonstrate a psychological approach.

A final perspective pertains to the psychological effects that certain environments may have on individuals, yielding particular states (Farshchi and Fisher, 2006); in the field of psychology, embodied cognition, which posits that cognition is shaped and influenced by the bodily experience of the environment, make radical propositions. It has been shown, for instance, that experiencing physical warmth promotes interpersonal relations (Williams and Bargh, 2008) and experiencing social inclusion can affect a judgment of temperature and desire for hot and cold experiences (Zhong and Leonardelli, 2008). Secondly, moral decisions have been shown to affect temperature perception (Taufik et al., 2015). Taken together, these findings suggest psychological factors can affect bodily sensations directly.

In the work presented here, we are interested in the overall experience of thermal comfort. Emotion psychology bridges the psychological antecedents of an event to the unfolding of psychological and physiological responses to that event. In the field of building design, adaptive comfort theory is the theoretical tradition that provides the most insight into psychological factors, and we therefore seek to enhance this understanding of occupants' experience with insight from psychology.

### Appraisal theory of emotion: factors that affect the evaluation of sensations

**Figure 2:**
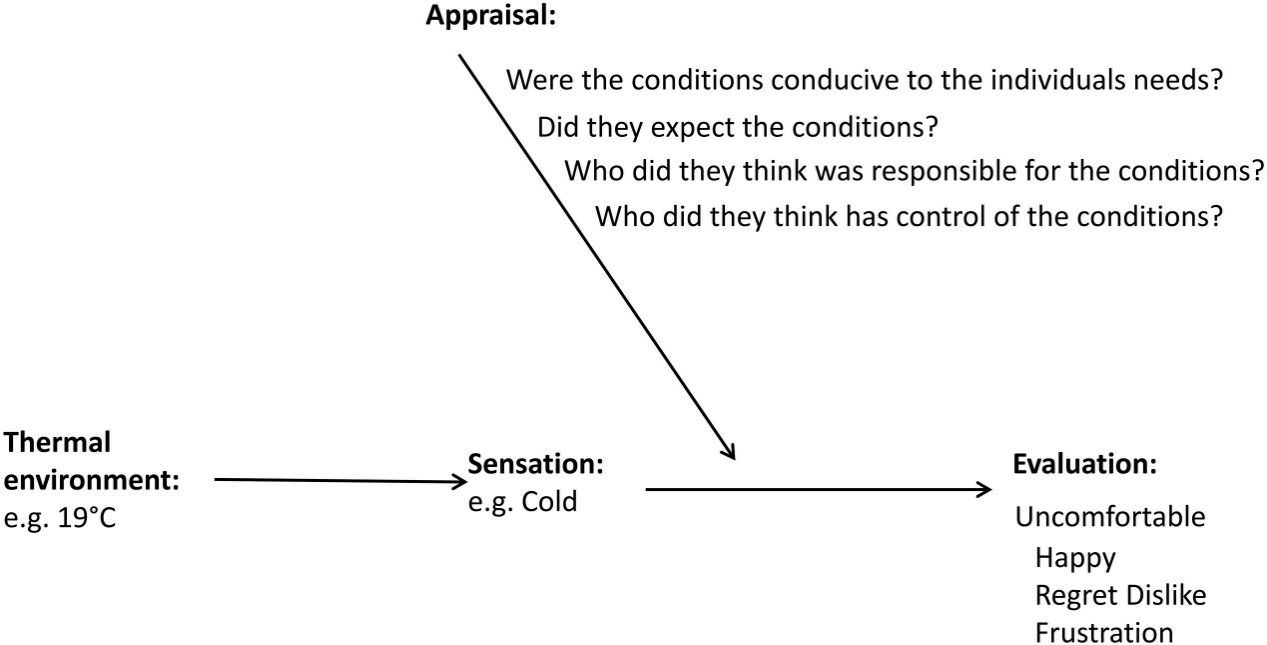
**Appraisals mediate how sensations are evaluated**.

A fundamental question in the field of emotion psychology concerns the fact that two people may be presented with the same situation and yet have different subjective experiences. A growing body of results suggests that the appraisal of the situation mediates the sensation and the ensuing evaluative response (Arnold, 1960; Scherer et al., 2001)—see Figure 2. It is this subjective appraisal process that influences and shapes the unique emotional response to a particular stimulus, giving rise to a wide range of emotions. In the context of thermal experience, two people can feel the same temperature, but evaluate the situation differently depending on whether it is appraised as conducive or obstructive to their respective needs. For instance, two people could be in a cold office, and one may feel happy because the temperature wakes them up and creates the optimal conditions for work, whereas another person could feel upset because the cold sensation disrupts their ability to focus.

The example above exposes the relationship between appraisals and the evaluation that follows. In this example, a single appraisal dimension of conduciveness is used to evaluate a thermal situation. One person appraises the situation as conducive to their goal and experiences positive emotions, while the other appraises the same environment as obstructive and thus experiences negative emotions. These appraisal processes occur at a subconscious level, which influences the overall experience. Appraisal theorists attempt to characterize the quality of relevant appraisal dimensions and make predictions for the ensuing emotions (Arnold, 1960; Scherer et al., 2001).

We propose to use appraisals as proxies for understanding how participants' past experience will affect their conceptualization of a given environmental stimulus or scenario. This experience is reduced to a limited number of fixed appraisals, and one of the simplest appraisals is whether a stimulus is consistent with a person's motives and desires or not; if it is, then the resulting emotion is likely to be positive, if not, then the emotion is likely to be negative.

Further appraisal dimensions can be used to predict which positive or negative emotions will be experienced. For instance, another common appraisal is what or who is responsible for the cause of the experience. If a person appraises that they are themselves responsible (for a negative outcome), the theory predicts they will experience regret. If someone else or unavoidable circumstances (e.g., the weather) are believed to be the cause, then anger, frustration, or resignation would be experienced. Together, these appraisal dimensions can help predict specific emotions (Scherer, 2001).

The value of appraisal theory for the field of building design is that it provides a framework to understand how people's conceptualization of a situation affects their experience. This sheds light on the mapping between sensation and evaluation. Four appraisals, which are implicit in adaptive comfort theory and explicit in appraisal theory, may be useful to our aim. These are goal conduciveness, causality for the situation, perceived control, and expectation (Smith and Ellsworth, 1985; Roseman, 1996; Scherer, 2001). Used together they predict a range of positive and negative emotions (Table 1). We suggest that these four appraisals are similar to concepts that have been found to be important to the adaptive theory of thermal comfort. In the next section, we draw on the above-mentioned theoretical traditions and review four hypotheses we formulated to explore the mediating effect of appraisals on the ensuing experience of thermal comfort.

**Table 1:** **Emotions mapped to different appraisal combinations (derived from Roseman, 1996; Scherer, 1999)**.

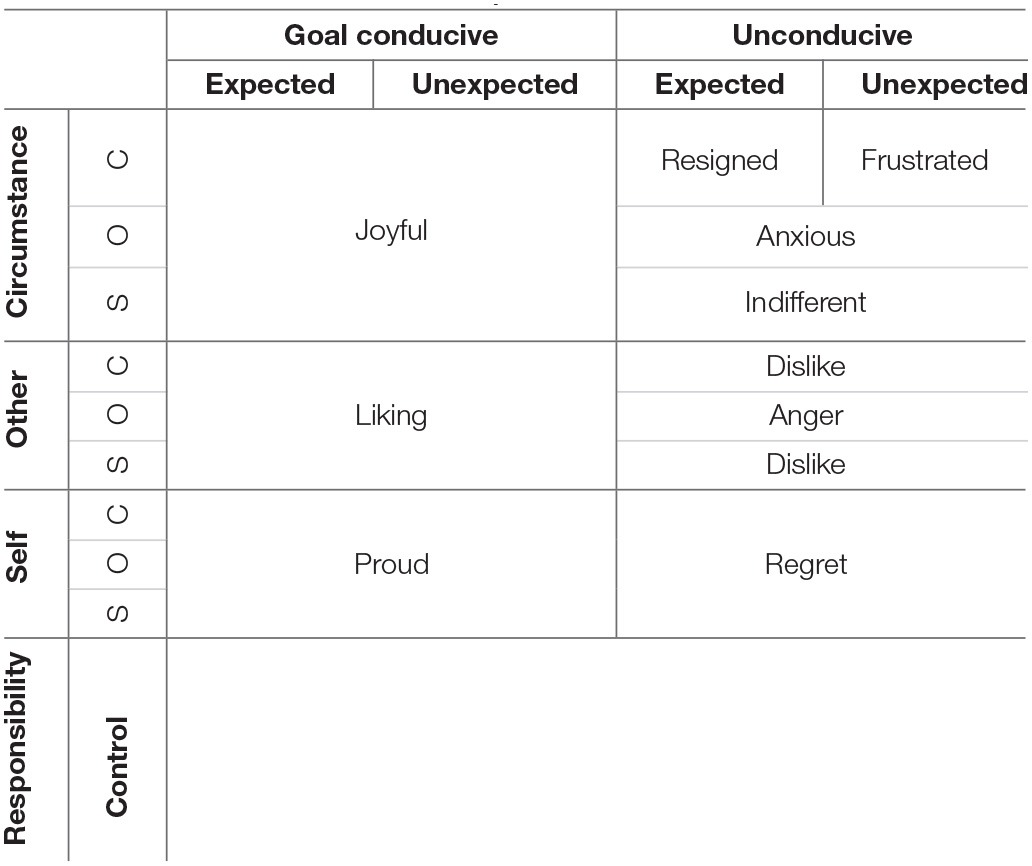

### Hypotheses

We focus our investigation on four psychological factors that are present in both Adaptive Comfort theory and the Appraisal theory of emotions, either implicitly or explicitly, and on related effects over thermal experience. The purpose of this comparison is to study the predictions from both sets of theories and highlight aspects that could be operationalized in design practice. The psychological factors of interest here relate to the information processing units that may serve in the evaluation of a given thermal environment by a given occupant. Although it is believed that such evaluations may be performed over continuous sets of criteria, we restrict our investigation to discrete, extreme situations to formulate working hypotheses.

Conduciveness—relates to the extent to which a given thermal event will serve or obstruct an occupant's goal. High conduciveness implies that the event supports the occupant's present goals, whereas low conduciveness implies that the event does not support or even hinders their goals in some ways.

Causality—relates to the extent to which a given thermal event has been caused by either unavoidable circumstances, the occupant themselves, or other occupants. By unavoidable circumstances, we mean natural conditions, e.g., a sunny day, or changes in the environment that affect occupants, e.g., a malfunctioning radiator. An example of a situation caused by the occupant themselves or others may be the opening of a window, or a voluntary change in the setting of the thermostat, in line with or against shared space policies (Leaman and Bordass, 2007).

Perceived control—relates to the extent to which the occupant perceives they have control over their environment (Brager and de Dear, 1998). This aspect is particularly relevant, because practical provisions are formulated in building design to recommend the number, type and access mode for such control interfaces. For obvious reasons, the amount of control available will vary depending on the environment, and we expect a wide distribution of responses.

Expectations—relates to the extent to which the occupant was expecting a given thermal event (Brager and de Dear, 1998; Ole Fanger and Toftum, 2002). High expectancy means that the occupant was expecting the event to occur, and low expectancy that they were not expecting it.

We thus formulate the following predictions, which drove the elaboration of our questionnaires and the ensuing analyses of the data. In our interpretation of the results, we compare the predictions from both sets of theories—see Table 2.

**Table 2:** **Summary of hypotheses**.

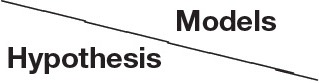	**Adaptive comfort theory**	**Appraisal theory of emotions (bodily symptoms)**	**Ensuing affect and emotions**
(1) Conduciveness	High → Comfortable	High → Neutral	High → Joy, pleasure
	Low → Uncomfortable	Low → Cold, hot	Low → Displeasure
(2) Causality	Circumstances → Comfortable	Circumstances → Neutral	Circumstances → Resignation, anxiety
	Others/Self → Uncomfortable	Others/Self → Too hot, too cold	Others/Self → Dislike, anger
(3) Perceived control	High → Comfortable	High → Neutral	High → Anger, anxiety
	Low → Uncomfortable	Low → Too hot, too cold	Low → Resignation, frustration
(4) Expectations	High → Comfortable	High → Neutral	High → Resignation
	Low → Uncomfortable	Low → Too hot, too cold	Low → Frustration

## Methodology

### Participants and buildings

As part of a wider field study focusing on evaluating the relationship between environmental factors and psychological experience, occupants of seven office buildings responded to our survey (*N* = 166). The wider field study consisted in the monitoring and recording of environmental factors during a typical work day. The set of buildings was constructed so as to offer a wide range of heterogeneous environments (open space, closed offices, etc.). The sample size is similar to that of other appraisal studies [Folkman and Lazarus (1985), *N* = 182; Roseman (1996), *N* = 136–189; Scherer and Ceschi (1997), *N* = 112]. Respondents were a range of ages and genders (Table 3) and from seven different buildings (Table 4). Participants were rewarded with a snack of their choice. The study was approved by the University of Reading Ethics Committee, in accordance with the Helsinki Declaration of 1975, as revised in 2000. Written consent was obtained from participants.

**Table 3:** **Summary of participants**.

***N***	**Female**	**Male**	**Undisclosed**	**18–34 years**	**35+ years**	**Undisclosed**
166	105	57	4	84	77	5

**Table 4:** **Overview of buildings**.

**Building**	***N* (% resp.)**	**Occupier**	**Typology**	**Plan**	**HVAC**
A	9 (18)	Design	Open plan	Shallow	MM
B	9 (69)	Academic	Open/Cell	Shallow	NV
C	46 (17)	Academic	Open/Cell	Shallow	NV
D	29 (15)	Academic	Open/Cell	Shallow	MM
E	9 (18)	Design	Open plan	Shallow	NV
F	25 (2)	Charity	Open plan	Deep	AC
G	39 (26)	Design	Open plan	Shallow	NV

*NV, naturally ventilated; MM, Mixed mode; AC, fully air conditioned*.

### Questionnaire development

Tapping into the subjective experience of an individual is a major challenge, because the mere attempt to ask a question is likely to disrupt the unfolding experience altogether. To eliminate this disruption, we chose to use a recall survey, in which participants were asked to recall a salient event in their recent past and to answer a number of questions about that event. This also allows us to access a much greater range of experiences than if it was necessary to be present at the time of the event, measuring the thermal environment as the experience unfolded. The reliance solely on user reported data, with little or no measurement of the physical nature of the stimuli, is common in psychology (Fontaine et al., 2007) and is appropriate here because of this study's focus on the relationship between participants' sensation and their evaluation.

The recall survey started with a prompt for the participants to recall an event in detail. To do this they were asked to:

“*Imagine a specific time when you have been aware of the temperature in your office and it has given rise to strong feelings. Describe what happened leading up to the event and how you felt.”*

After this, a number of questions were asked about each of the four appraisal dimensions. Details of the questions and how they were combined can be found in the Appendix in Supplementary Material. These were used to understand:

- Whether the participant felt the event was conducive to them (appraisal 1);
- Who or what they thought caused the event (appraisal 2);
- Who or what they thought controlled conditions in their office (appraisal 3);
- How much they had expected the event to happen (appraisal 4).

To finish the survey, there was an open response to describe feelings and a closed list of emotions to choose from: frustrated, resigned, dislike, indifferent, angry, anxious, liking, joyful, regretful, proud, or none of these. Then three questions were asked about the participant's thermal experience, using a thermal sensation scale, a comfort scale and an acceptability scale.

### Analyses

We examined whether appraisals have an effect upon emotions, acceptability, comfort, and sensation. The model used compares the likelihood of a particular evaluation, dependent upon the score on an appraisal dimension. The most appropriate statistical model for this is a logistic regression model. This allows prediction of the presence or absence of a given factor (a set of emotions or acceptance) dependent upon an ordered factor (the appraisal dimension). An extension to this model is the ordinal logistic model, which predicts the likelihood of achieving a given level of comfort or sensation depending on an appraisal dimension.

Equation (1) shows the logistic regression model. The model comprises a linear function and a link function. In the same way as standard linear models, the coefficients are derived so as to maximize the fit of the model. The link function *m*() transforms the linear model to a probability of success, π_*i*_ bounded between one and zero. There are several functions that fit this criteria, the most commonly used are the “logit,” “probit,” “cauchit,” “log,” and the “complementary log log” (McCullagh and Nelder, 1989). In this study we compared all possible link functions and selected the best fitting model.

(1) $$\begin{array}{r} \pi_{i}\;=\;m\left(\beta_{0}+\beta_{1}x_{i}+\beta_{2}x_{i}+\ldots \right) \end{array}$$

To compare logistic models, we used a chi square test of the deviance accounted for by the regression model. For both the logistic and ordinal logistic model we also characterized the model by the likelihood that the regression coefficients (β_*i*_) are non-zero.

## Results

### The experiences reported

#### Sensation, comfort, and acceptability

Participants were asked to report their thermal experience during the period that they recalled. Generally, they recalled periods of time when they were experiencing extreme thermal sensations, either too hot or too cold (Table 5). Most participants found this to be *uncomfortable* rather than *very uncomfortable* (Table 6). These conditions were found to be unacceptable by the majority of participants (Table 7).

**Table 5:** **Thermal sensation counts**.

**Thermal sensation**	**Count**
Cold	30
Cool	6
Slightly cool	1
Neutral	8
Slightly warm	6
Warm	30
Hot	84
Undisclosed	1

**Table 6:** **Comfort counts**.

**Comfort rating**	**Count**
Very uncomfortable	42
Uncomfortable	84
Slightly uncomfortable	38
Comfortable	1
Undisclosed	1

**Table 7:** **Acceptability counts**.

**Acceptability rating**	**Count**
Not acceptable	129
Acceptable	33
Undisclosed	4

#### Emotions recalled

**Figure 3:**
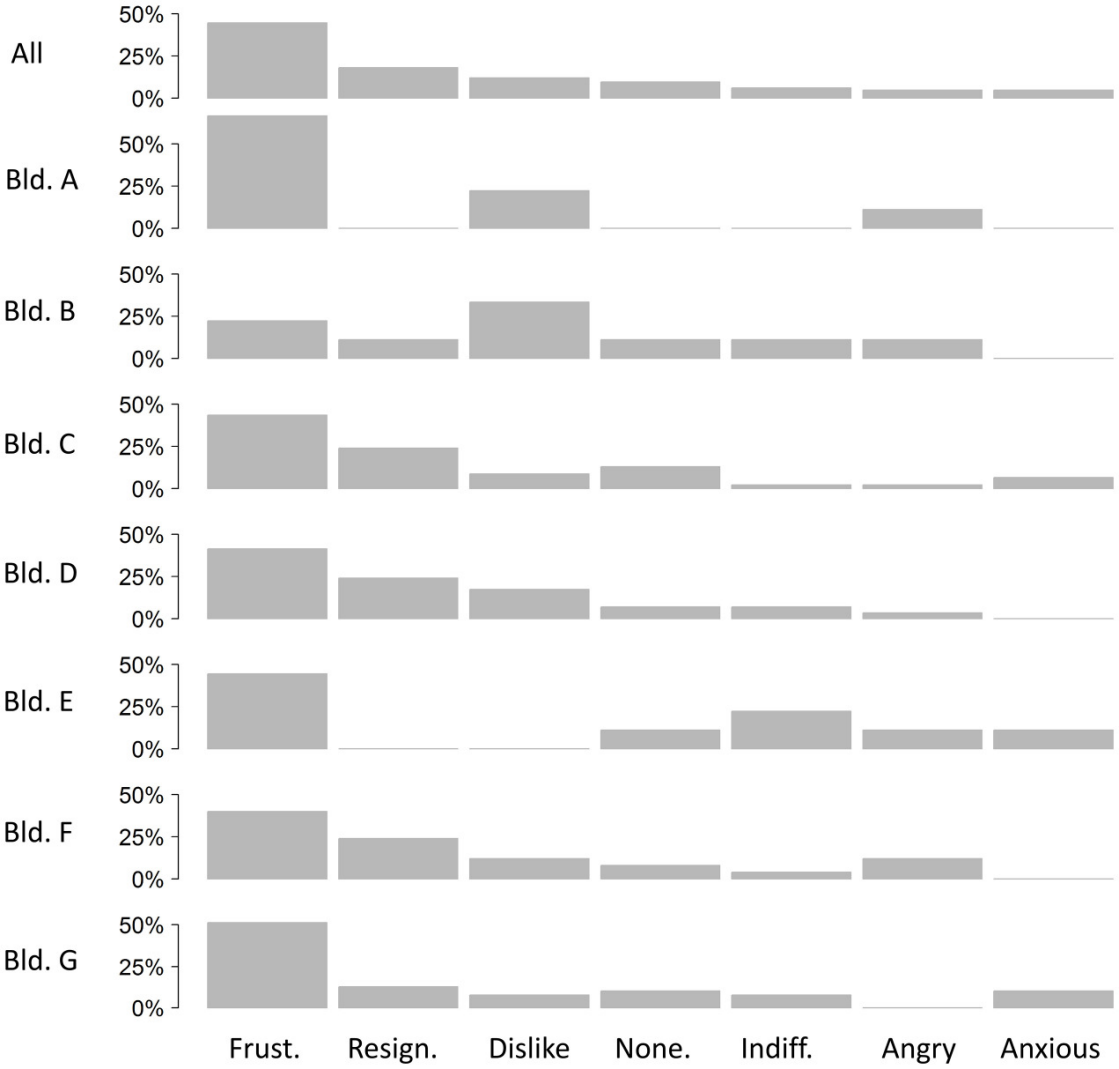
**The emotions reported across all buildings**.

Participants were asked to choose one of several emotions that best matched their feelings from a closed list. No one reported a positive emotion or an emotion associated with personal responsibility, i.e., regret (Table 8). Mostly, participants reported feeling frustrated, resigned, or a dislike of the situation. A smaller number of participants felt indifferent, angry, or anxious. There were also sixteen participants who felt that none of the ten emotions fitted well with how they felt. Across buildings, the trend was generally the same, except Buildings A and B where people were more likely to feel dislike and building F where they were more likely to feel angry (Figure 3).

**Table 8:** **The emotions reported across all buildings**.

**Emotion**	**Count**
Frustrated	74
Resigned	30
Dislike	20
None of these	16
Indifferent	10
Angry	8
Anxious	8
Liking	0
Joyful	0
Regretful	0
Proud	0

### The appraisals

**Figure 4:**
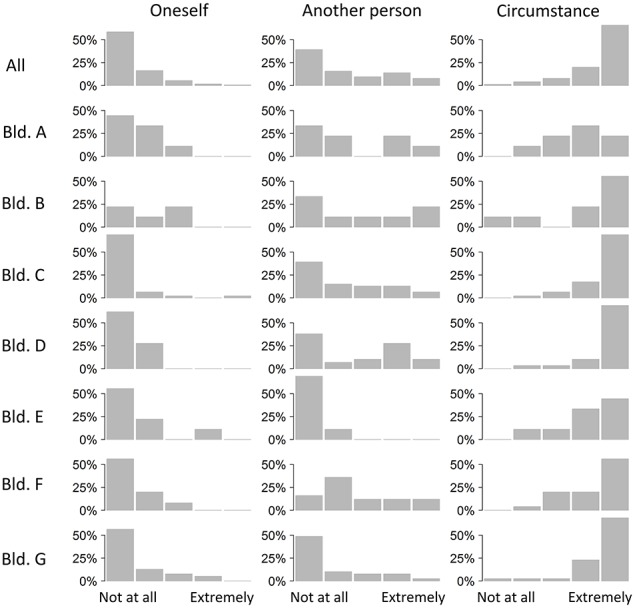
**Who is appraised as responsible for the event, across the different buildings**.

**Figure 5:**
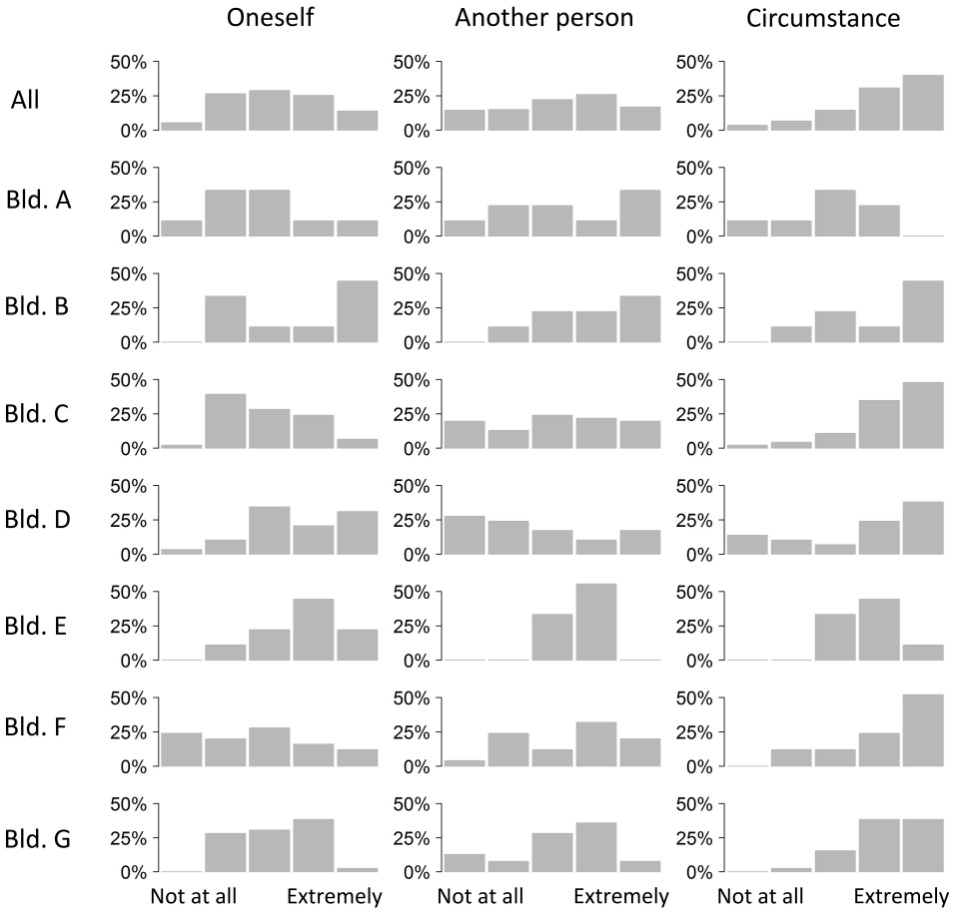
**Who is appraised as in control in general, across the different buildings**.

**Figure 6:**
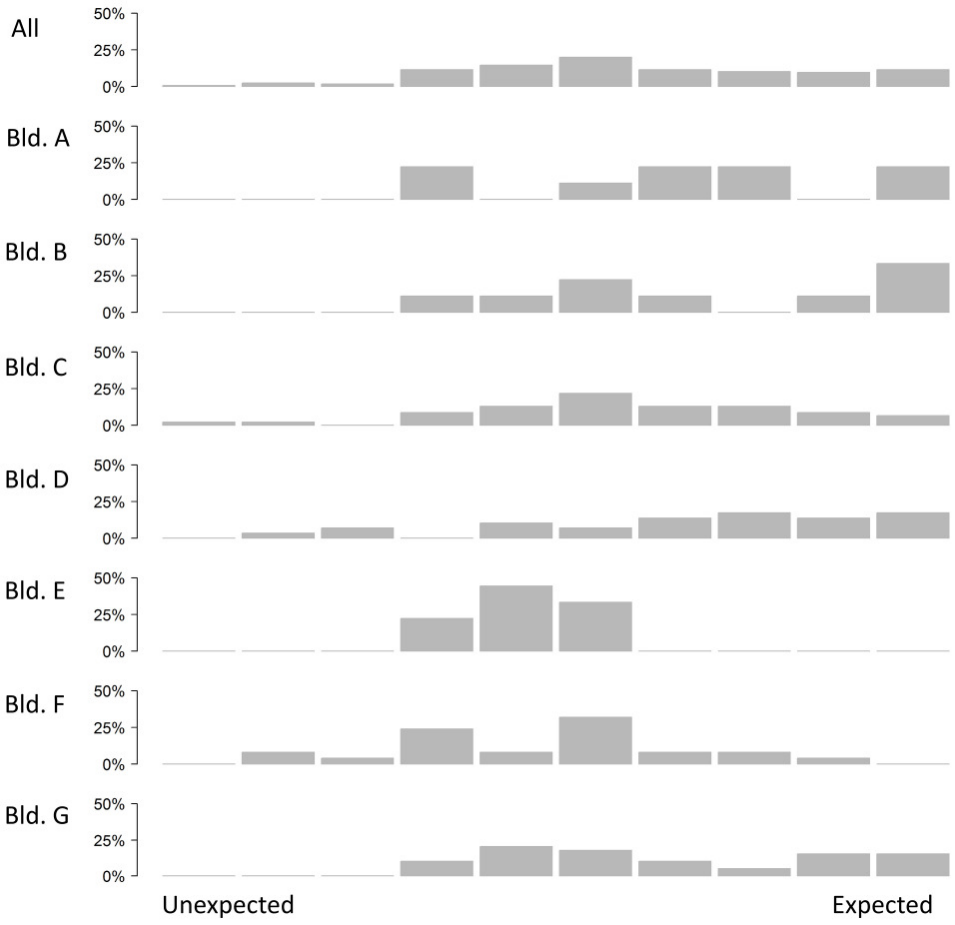
**Appraisal of expectedness of the event, across different buildings**.

Generally, participants reported that the event was unpleasant and worsened their ability to work. We also asked who they thought was responsible for the events leading up to their emotional experience (Figure 4). They rarely thought they themselves were responsible. We asked the participants who they thought was generally in control of the temperature in their office. Occupants of building F especially felt they had little control. Occupants of buildings C and D thought no person was in control. Across most buildings circumstances were thought to control conditions (Figure 5). Overall, there was a mixture of whether people thought the event they reported could have been expected. However, there is a lot of difference between buildings. Occupants from buildings E and F tended to report events that were unexpected. Elsewhere events reported had been expected (Figure 6).

### Using appraisals to predict emotions

**Figure 7:**
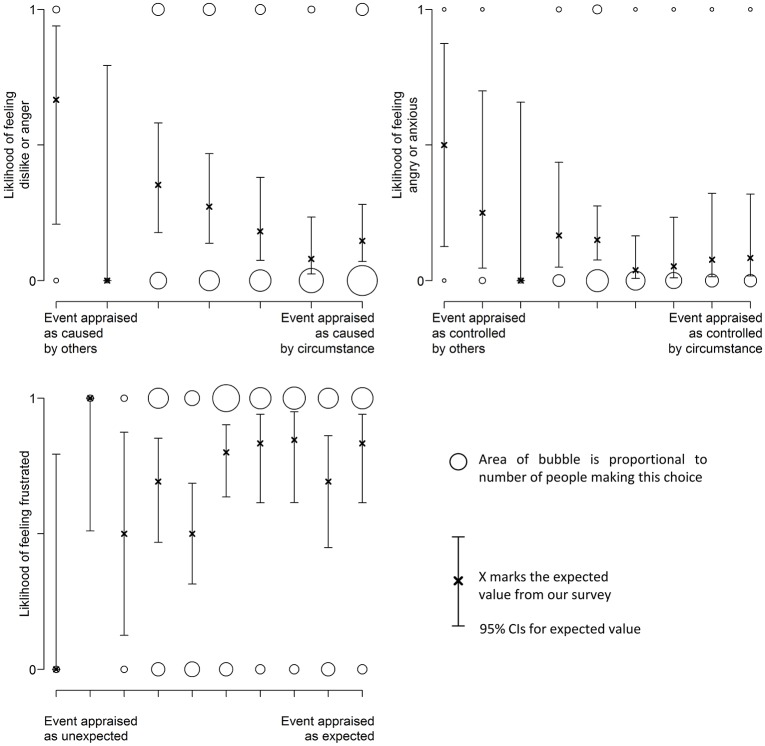
**Appraisals of responsibility and control have an effect on the emotion reported**.

The absence of positive emotions and the absence of positive appraisals of conduciveness is in accordance with appraisal theory. However, the lack of positive emotions also means it is difficult to build a comprehensive statistical model for validation. For the remaining three appraisals, the emotions reported were partitioned into two groups according to the relevant hypothesis, i.e., for Causality, one group was aligned with the appraisal of caused by another (dislike and angry) and the other with appraisal of caused by circumstance (frustrated, resigned, indifferent, anxious). Figure 7 shows how the likelihood of feeling one set of emotions rather than another varies with participants' appraisal.

We tested several link functions to model these data, and report statistical tests of the best model in Table 9. These suggest that there is a tendency to feel angry or dislike when another person is deemed responsible for the thermal experience, in support of predictions from appraisal theory. The results also show a tendency to feel angry or anxious when another person is appraised as being in control of the thermal experience, again supporting appraisal theory. For the appraisal of Expectations, there is not such an obvious pattern as for the other appraisals.

**Table 9:** **Characteristics for emotions models**.

**Appraisal**	**Best link function**	**χ**^2^ **goodness of fit**		**Model coefficients**	
		**χ^2^**	**Single tailed**	**β_0_**	**β_1_**
Responsibility	Poisson	7.1 (*df* = 1)	*P* = 0.01	−1.1 (*p* < 0.001)	−0.28 (*p* = 0.003)
Control	Cauchit	3.8 (*df* = 1)	*P* = 0.05	−2.7 (*p* < 0.001)	−0.65 (*p* = 0.03)
Expectation	Cauchit	2.70 (*df* = 1)	*P* = 0.10	−0.15 (*p* = 0.81)	0.21 (*p* = 0.11)

### Using appraisals to predict comfort and acceptability

**Figure 8:**
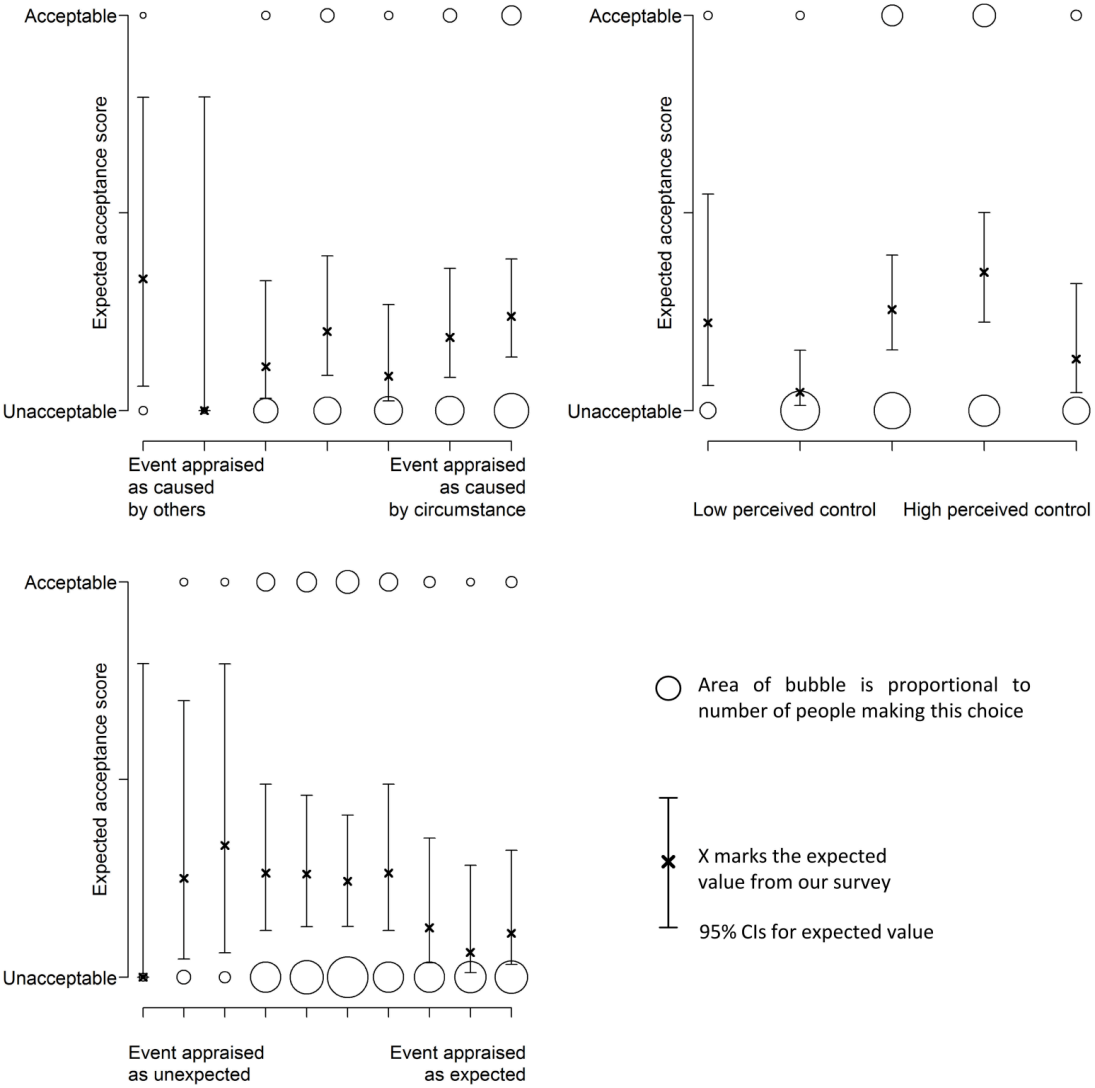
**The appraisal of expectation has a small effect on acceptability**.

**Figure 9:**
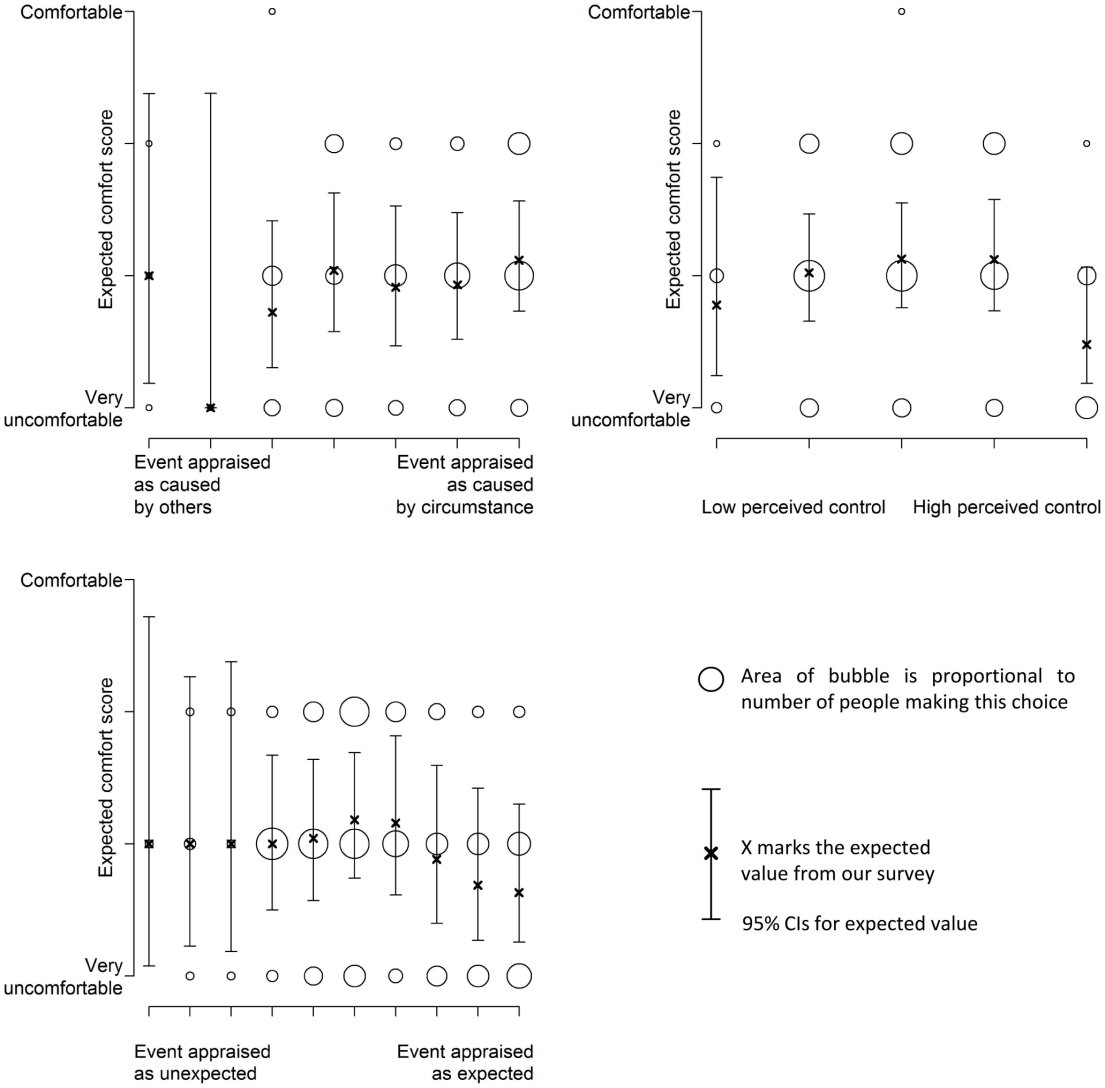
**The appraisals of control and expectation have a small effect on comfort**.

Figure 8 shows how the likelihood of finding a thermal experience acceptable varies with participants' appraisal. Figure 9 shows the effect of the same appraisals on comfort rating. Again, we tested several link functions to model the data, statistical tests of the best models are reported in Table 10. These suggest that the appraisals have little effect on the acceptability of the experience. There is a weak link that suggests that the more a situation is expected, the less acceptable it is. Similar results are found for comfort (Table 11).

**Table 10:** **Characteristics of acceptability models**.

**Appraisal**	**Best fitting link function**	**χ**^2^ **goodness of fit**		**Model coefficients**	
		**χ^2^**	**Single tailed**	**β_0_**	**β_1_**
Responsibility	Cauchit	0.82 (*df* = 1)	*P* = 0.37	−2.2 (*p* < 0.01)	0.24 (*P* = 0.36)
Perceived control	Probit	2.46 (*df* = 1)	*P* = 0.12	−1.3 (*p* < 0.001)	0.16 (*P* = 0.11)
Expectation	Probit	3.48 (*df* = 1)	*P* = 0.06	−0.27 (*p* = 0.39)	−0.10 (*P* = 0.06)

**Table 11:** **Characteristics for comfort models**.

**Appraisal**	**β_1_**	***SE***	***t*-Value**	***p*-Value**
Responsibility	0.18	0.10	1.76	0.08
Perceived control	–0.20	0.13	–1.55	0.12
Expectation	–0.16	0.07	–2.26	0.02

### Using appraisals to predict deviation from neutral sensation

**Figure 10:**
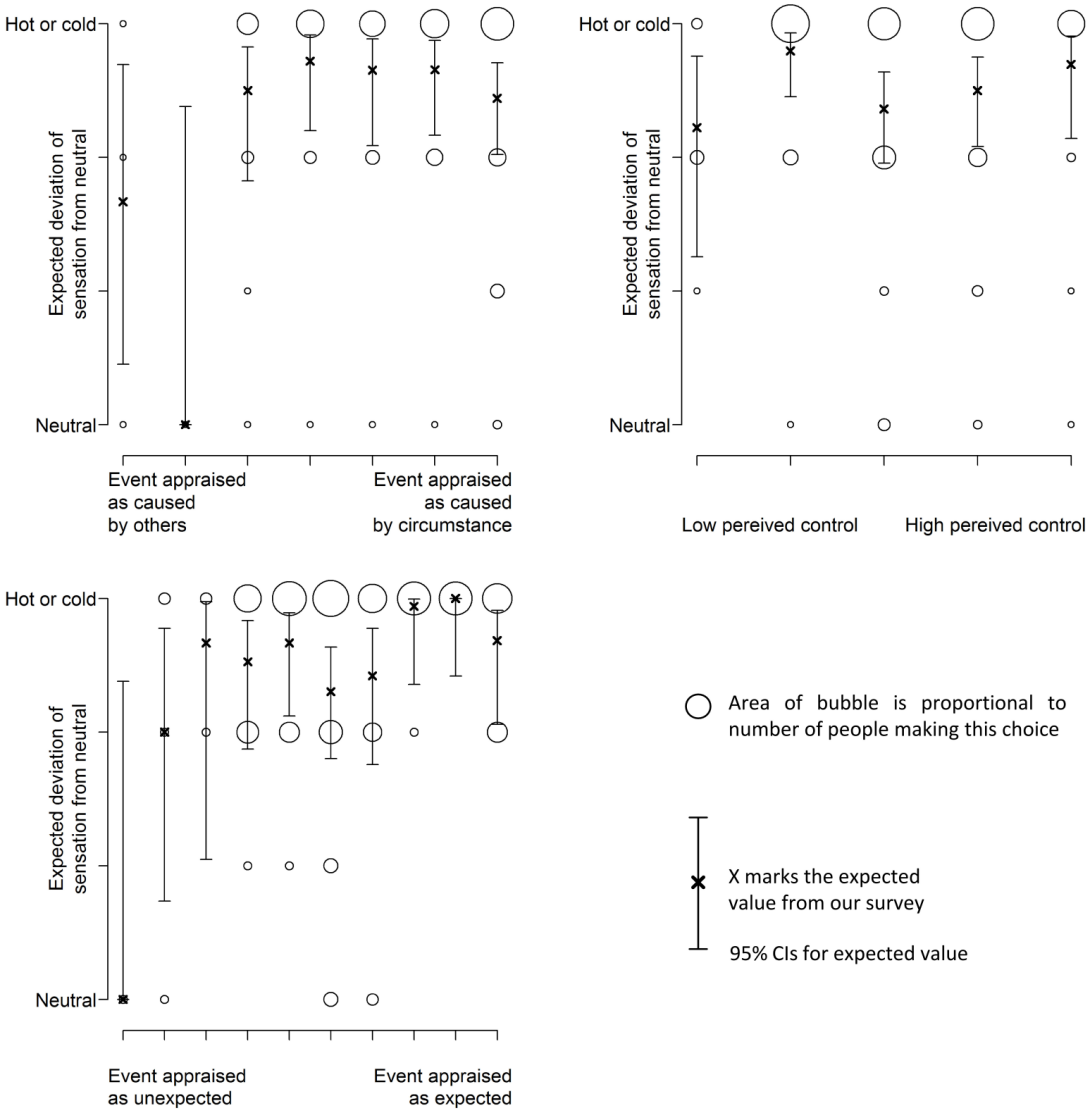
**The appraisal of expectation has an effect on thermal sensation**.

Figure 10 shows how the likelihood of reporting a neutral thermal sensation changes with participants' appraisal.

Table 12 shows the different model characteristics. These results suggest that the expectation appraisal influences bodily symptoms (thermal sensation) but the other appraisals do not.

**Table 12:** **Characteristics for sensation models**.

**Appraisal**	**β_1_**	***SE***	***t*-Value**	***p*-Value**
Responsibility	0.04	0.12	0.30	0.76
Perceived control	0.07	0.15	0.51	0.61
Expectation	0.24	0.87	2.75	0.006

## Discussion

Taken together, our results show that appraisal processes are important for shaping evaluation of the thermal environment. This approach complements adaptive comfort theory of thermal experience in building design. This work supports the notion that thermal experience is rich and complex, and requires understanding of how people conceptualize their thermal environment (Heschong, 1979). It is possible that appraisals, especially Conduciveness, could be driven by the thermo-physiological state of the participant, though there is no need for this to be the case.

The appraisals of Causality and Control were less useful for predicting the traditional thermal comfort evaluations of acceptability and comfort. This contradicts the extensive literature on perceived control and thermal comfort (Brager and de Dear, 1998; Hellwig, 2015). This unexpected result could be a side effect of the recall method. The recall method provides access to thermal events that are of high saliency, thereby biasing the distribution of events under investigation. It is possible that perceived control does not affect the severity of the most extreme bad events, or rather, that the skewed distribution of events reported did not allow for our models to explore the full range of responses.

The appraisal of Expectations was successful in predicting comfort evaluation. However, the correlation was opposite to that expected. The more an event was predictable or expected the more uncomfortable it was. Thermal comfort theory would predict that occupants acclimatize to events over time (Brager and de Dear, 1998). Our results suggest that events that are novel and fleeting may cause less discomfort than recurring and predictable problems.

When we asked about Expectations, participants may have focused on recurrent salient situations, whereas the classic expectation of thermal comfort refers to repeated and continuous exposure to a ubiquitous climatic experience. Given this observation, it appears that our results draw attention to a different type of expectation effect. Namely that when problematic conditions are recurrent, they become less and less acceptable.

The work on psychological adaption and embodied cognition hints at two different mechanisms through which psychological factors could affect thermal experience. The first mechanism suggests that psychological factors change the mapping between thermal sensation and thermal evaluation. These theories suggest that the benefit of personal control is that it reduces stress from mildly unfavorable conditions and effective control provides pleasure (Hellwig, 2015). In contrast, embodied cognition suggests that the psychological factor would change thermal sensation itself. Interestingly, a study carried out in a climate chamber by Zhou et al. (2014) suggests that perceived control actually changes bodily sensation as well as reducing stress.

Our results support theories of embodiment because where appraisals have an effect on comfort, they also have an effect on reported sensation. However, this can only be taken as weak support for embodiment because our field study did not assess specific thermal environments. The inclusion of synchronous temperature measurements would provide conclusive evidence that the appraisal caused a sensation change, as opposed to thermal sensations causing both comfort and appraisal.

The lack of positive emotions supports work that suggests that temperature is a hygiene or basic factor responsible only for dissatisfaction (Herzberg, 1964; Kim and de Dear, 2012). However, it may be that people chose to focus just on negative events from their past—although they were not probed to do so. Future investigations could be contrived to test this by asking participants to describe two experiences and stipulate that one had to be positive. This approach would provide a greater range of experiences and hopefully contribute positive emotions to improve our analyses.

To improve the method and repeatability, the survey could also be made easier to analyze. We focused here on the comparison of sets of theories and assessment of the best explanatory models. Further studies may choose to simplify the design by focusing on particular aspects. First, appraisal dimension could be specified to ease coding. The current system of combining many ordinal responses is convoluted and builds in uncertainty, which was reflected in our analyses. Second, continuous response for variables could be used. This would mean that analysis could be done with genuine ratio scale numbers rather than an ordinal scale that was transformed into a ratio scale.

## Conclusions

Appraisal theory provides a simplified way to encapsulate people's thoughts about their thermal experience. These thoughts cover not only a person's core temperature and peripheral thermal stimulus but also their past experiences and future desires. The theory does not try to predict why people make certain appraisals but it identifies which appraisals are key. Overall, our results show that it is the combination of these appraisals that shapes a thermal experience. Multidimensional appraisals require multidimensional evaluations, and in this case we have successfully used ten emotions to describe thermal experience.

Our analysis suggests a new aspect to how expectation affects psychological adaption. We observe that recurrent problems (those that happened often and were predictable) resulted in greatest discomfort. People did not appear to adapt to them. This suggests an alternative way to conceptualize expectation.

With further modifications, the survey developed here could be used as a diagnostic tool where discomfort and dissatisfaction are caused because of psychological factors (as opposed to poor thermal conditions). From this it may be possible to design a program of measures that tackle those psychological causes. This would be in contrast to current industry approaches that focus on costly technical fixes and chase ever more control over the physical environment.

Interpretation, meaning, and other psychological approaches have been shown to play a part in subjective experience across a range of indoor environmental quality indices (Kwon et al., 2011; Lehman, 2011). This leads us to suspect that this method could be used to understand emotions and their appraisals caused by multisensory experiences of buildings, beyond just thermal comfort.

## Author contributions

The study is based on TK's EngD research work. TK was responsible for the research design, data collection, and analyses. DC and ER provided invaluable guidance and supervision respectively, throughout the research. All authors contributed to the production of this manuscript.

## Funding

This work would not have been possible without the Institute of Technology for a Sustainable Built Environment (TSBE), Reading University, the Engineering and Physical Science Research Centre (EPSRC) Doctoral Training Scheme (grant number EP/G037787/1) and TK received additional funding from BuroHappold Engineering for the duration of his engineering doctorate.

### Conflict of interest statement

The authors declare that the research was conducted in the absence of any commercial or financial relationships that could be construed as a potential conflict of interest.
