# Supplementary material for: Cognitive Appraisals Affect Both Embodiment of Thermal Sensation and Its Mapping to Thermal Evaluation
Source: Front Psychol. 2016 Jun 27;7:800. doi: 10.3389/fpsyg.2016.00800 (PMC4921490; doi:10.3389/fpsyg.2016.00800)
Supplement: Supplementary file 1 [file DataSheet1.docx]

**Appendix II: Combination of independent variables**

This section summarises how individual survey questions were combined into scores for each appraisal dimension. This was not done for conduciveness because of the results obtained.

For the appraisal of responsibility the participant’s seven relevant answers were grouped in to those that suggested a circumstantial cause (i.e. building or weather) and those that suggest a cause by another person (i.e. colleague or building/facilities manager).Those that suggested a cause by self were discarded. Each of the two groupings were combined into a single score by taking the maximum rating across the group (i.e. extremely equals highest possible score). We took the maximum across the questions instead of an average because the emotional response is contingent on the relative importance of the highest scoring items in each group (i.e. overall did this participant think the event was more caused by a person, regardless of the specific person, or more caused by circumstance, regardless of the specific circumstance). These two groupings were combined into a single score by subtracting the person appraisal from the circumstance appraisal.

$$A_{2}=\max\left( Q10,Q11,Q12,Q13 \right)-max(Q8,Q9)$$

For the appraisal of control the nine relevant answers were combined into three scores (circumstance, self and other) by taking the maximum rating across the relevant answers. The circumstance and self groupings were combined into a single score by subtracting the person appraisal from the circumstance appraisal, this was used to test hypotheses about control and emotions.

$$A_{3}=\max\left( Q22,Q23,Q24 \right)-max(Q19,Q20,Q21)$$

Those that suggested perceived control were separated and used for testing hypotheses about control and thermal comfort.

$$A_{3perceived control}=\max\left( Q18,Q25,Q26 \right)$$

Producing a combined expectation score, from the four relevant questions, was more complicated than the other appraisal scales. Firstly the two questions “How often does it happen in winter?” and “How often does it happen in summer?” were merged by taking the highest score as per before. This produces a score equivalent to, does it happen often in any season. This was then combined with the levels for the two remaining questions by adding scores that were suggestive of predictability and subtracting scores associated with suddenness. This produced a scale running from -3 (most unexpected) to 13 (most expected).

$$A_{4}=\max\left( Q16,Q17 \right)+Q15-Q14$$
